# Trends towards integrated long-term care for older people in Europe—Harnessing the knowledge base

**Published:** 2012-09-04

**Authors:** Kai Leichsenring

**Affiliations:** European Centre for Social Welfare Policy and Research, Berggasse 17, A-1090 Vienna, Austria

**Keywords:** INTERLINKS, integrated care, long-term care, older people

## Abstract

INTERLINKS (“Health systems and long-term care for older people in Europe: modelling the interfaces between prevention, rehabilitation, quality of services and informal care”), was a project co-financed by the EU 7th Framework Programme (Grant agreement no. 223037) with a purpose of constructing and validating a framework to describe, analyse and improve long-term care (LTC) systems for older people from a European perspective. The project was co-ordinated by the European Centre for Social Welfare Policy and Research (Vienna) and carried out by 13 research agencies from 15 European countries. The three-year project was completed in December 2011. The ultimate outcome of the project is the INTERLINKS Framework for Long-Term Care that defines themes, sub-themes and 135 key-issues to be addressed when striving for an integrated system. The Framework has been translated into an interactive website, where key-issues are illustrated by practice examples from the participating countries and beyond.

Underpinning this work were in-depth European reviews and conceptual discussion papers by partners as well as a validation process of the Framework by representatives of national and European experts, and a peer-review of practice examples. At the end of the project period, it is important to promote INTERLINKS as an interactive and sustainable site for the EU community interested in research, practice and policies addressing the well-known shortcomings at the interfaces between health and social care.

This workshop will briefly present the project and give an overview of the themes, subthemes and key issues and their accompanying developmental processes. The audience will gain insight into current trends towards integrated long-term care for older people, based on the findings of INTERLINKS partners and innovative transversal themes from practice examples that have been identified across Europe.

The main focus of the workshop will be to discuss the evidence and knowledge base identified by the project: What does evidence mean in integrated long-term care? How could the web-based tool developed by INTERLINKS become a real platform for the discussion on issues to promote, assess and learn from European initiatives that strive to realise integrated long-term care for older people? What do we need to really learn from each other, not only across the health and social care divide, but also across national boundaries?

Powerpoint presentation available at http://www.integratedcare.org at congresses – San Marino – programme.

